# Clinical characteristics of patients with spinocerebellar ataxias 1, 2, 3 and 6 in the US; a prospective observational study

**DOI:** 10.1186/1750-1172-8-177

**Published:** 2013-11-13

**Authors:** Tetsuo Ashizawa, Karla P Figueroa, Susan L Perlman, Christopher M Gomez, George R Wilmot, Jeremy D Schmahmann, Sarah H Ying, Theresa A Zesiewicz, Henry L Paulson, Vikram G Shakkottai, Khalaf O Bushara, Sheng-Han Kuo, Michael D Geschwind, Guangbin Xia, Pietro Mazzoni, Jeffrey P Krischer, David Cuthbertson, Amy Roberts Holbert, John H Ferguson, Stefan M Pulst, SH Subramony

**Affiliations:** 1 Department of Neurology and McKnight Brain Institute, University of Florida, 1149 S. Newell Dr., L3-100, Gainesville, FL 32611, USA; 2 Department of Neurology, University of Utah, Salt Lake City, UT, USA; 3 Department of Neurology, UCLA, Los Angeles, CA, USA; 4 Department of Neurology, University of Chicago, Chicago, IL, USA; 5 Department of Neurology, Emory University, Atlanta, GA, USA; 6 Department of Neurology, Massachusetts General Hospital, Harvard Medical School, Boston, MA, USA; 7 Departments of Radiology, Neurology, and Ophthalmology, Johns Hopkins University School of Medicine, Baltimore, MD, USA; 8 Department of Neurology, University of South Florida, Tampa, FL, USA; 9 Department of Neurology, University of Michigan, Ann Arbor, MI, USA; 10 Department of Neurology, University of Minnesota, Minneapolis, MN, USA; 11 Department of Neurology, Columbia University, New York, NY, USA; 12 Department of Neurology, UCSF, San Francisco, CA, USA; 13 Data Management Coordinating Center, University of South Florida, Tampa, FL, USA; 14 ORDR-NCATS, NIH, Bethesda, MD, USA

**Keywords:** Spinocerebellar ataxia, Natural history, SARA, Progression rate

## Abstract

**Background:**

All spinocerebellar ataxias (SCAs) are rare diseases. SCA1, 2, 3 and 6 are the four most common SCAs, all caused by expanded polyglutamine-coding CAG repeats. Their pathomechanisms are becoming increasingly clear and well-designed clinical trials will be needed.

**Methods:**

To characterize the clinical manifestations of spinocerebellar ataxia (SCA) 1, 2, 3 and 6 and their natural histories in the United States (US), we conducted a prospective multicenter study utilized a protocol identical to the European consortium study, using the Scale for the Assessment and Rating of Ataxia (SARA) score as the primary outcome, with follow-ups every 6 months up to 2 years.

**Results:**

We enrolled 345 patients (60 SCA1, 75 SCA2, 138 SCA3 and 72 SCA6) at 12 US centers. SCA6 patients had a significantly later onset, and SCA2 patients showed greater upper-body ataxia than patients with the remaining SCAs. The annual increase of SARA score was greater in SCA1 patients (mean ± SE: 1.61 ± 0.41) than in SCA2 (0.71 ± 0.31), SCA3 (0.65 ± 0.24) and SCA6 (0.87 ± 0.28) patients (p = 0.049). The functional stage also worsened faster in SCA1 than in SCA2, 3 and 6 (p = 0.002).

**Conclusions:**

The proportions of different SCA patients in US differ from those in the European consortium study, but as in the European patients, SCA1 progress faster than those with SCA2, 3 and 6. Later onset in SCA6 and greater upper body ataxia in SCA2 were noted. We conclude that progression rates of these SCAs were comparable between US and Europe cohorts, suggesting the feasibility of international collaborative clinical studies.

## Background

Spinocerebellar ataxias (SCAs) are a group of autosomal dominant disorders of motor coordination caused by degeneration of the cerebellum and its afferent or efferent pathways. Among them, SCA1, 2, 3, 6, 7 and 17, and dentatorubral pallidoluysian atrophy are caused by an expansion of a polyglutamine (polyQ)-coding CAG repeat within the respective genes [1-3]. Although these SCAs are all rare, with an estimated prevalence of less than 4/100,000 for each SCA [4-8], SCA1, 2, 3 and 6 are the most common among all SCAs. Currently available data suggest that these SCAs are caused by a toxic gain of function by the expanded polyQ in the context of different proteins coded by the respective genes. Although SCA1, 2, 3 and 6 are known to cause progressively severe disability and often premature death, there are no effective treatments. However, the increasing understanding of the pathogenic mechanisms and development of new drug development technologies have raised hopes for novel treatments. The knowledge of natural history and availability of cohorts of clinically characterized research subjects are essential for well-designed clinical trials of these SCAs.

Cross-sectional studies have provided characterization of clinical phenotypes of SCA1, 2, 3 and 6 [1-3]. European investigators have studied the natural history of SCA1, 2, 3 and 6 by obtaining scores of the Scale for the Assessment and Rating of Ataxia (SARA) [9,11] and SCA Functional Index (SCAFI) [12,13]. For a parallel effort in the US, we have established the Clinical Research Consortium for Spinocerebellar Ataxias (CRC-SCA) as one of the 19 Rare Disease Consortia (RDC) of the NIH Rare Disease Clinical Research Network (RDCRN) [14] to investigate the natural history of SCA1, 2, 3 and 6 in 2009. The use of the same measures as those used in the European studies allows us to directly compare the data in two different patient populations across the Atlantic.

## Methods

### Patients

In this prospective study, subjects were recruited from ataxia clinics at 12 participating centers of the CRC-SCA at Columbia University, Emory University, Massachusetts General Hospital, Johns Hopkins University, University of California Los Angeles, University of California San Francisco, University of Chicago, University of Florida, University of Michigan, University of Minnesota, University of South Florida and University of Utah. At each center, study subjects were consecutively recruited from patients visiting the clinic specialized in ataxia or movement disorders. The patients seen in the clinic were referred by patients themselves, community physicians, local support groups and the National Ataxia Foundation. We also utilized the RDCRN Contact Registry to recruit study subjects in the catchment region. Under a uniform protocol approved by local IRBs, written informed consent was obtained from each patient. The inclusion criteria were 1) DNA diagnosis of SCA1, 2, 3 or 6 in the study subject or his/her affected family member(s), 2) phenotype consistent with the DNA diagnosis, 3) willingness to participate in the study, AND 4) the age of 6 years and older. The exclusion criteria were 1) known recessive, X-linked or mitochondrial ataxias, 2) concomitant disorder(s) that affect SARA and other ataxia measures used in this study, OR 3) exclusion of SCA1, 2, 3 and 6 by DNA testing.

### Genetic evaluation

After the enrollment, demographic data were obtained, and all patients were asked to provide DNA samples for SCA genotyping. DNA was extracted from blood leukocytes from patients; however, the participation in the genotyping study was not requirement for participating in the natural history study. The genotyping was performed in Dr. Stefan Pulst’s laboratory at University of Utah using multiplex PCR followed by capillary electrophoresis with internal standards to confirm the reports of commercial DNA testing. For quality control two CEPH DNA samples (1332-02 and 1347-02) were included in every run, and re-genotyping and Sanger sequencing were performed in 10% of samples.

### Clinical evaluation

The study started in April 2010 after all participating sites received IRB approval. At the baseline visit, neurological examination, SARA, functional stage (physician’s assessment of the patient’s overall function), timed 25-ft walk test (T25FW), 9-hole pegboard test (9HPT), the Unified Huntington’s disease Rating Scale functional assessment (UHDRS IV), EQ-5D (quality of life) visual analogue scale (EQVAS), Patient Global Impression, and Physician Global Impression were completed. We also estimated an abbreviated SCA Functional Index (SCAFI-AB) score, a version of the SCAFI [12] which includes the T25FW and 9HPT but not the PATA rate [12]. We did not include the PATA rate because of the large data variability in our pilot study (data not shown). The age at the baseline visit and the age at onset, which was defined as the age when the patient first noted gait ataxia during walking in ordinary circumstances, were recorded. Results were electronically entered in the case report form prepared by the CRC-SCA and the RDCRN Data Monitoring Coordinating Center (DMCC) and stored in the CRC-SCA Natural History Database at DMCC. The clinical evaluation was performed at the baseline visit and every six months thereafter until two years from the baseline visit or until the end of August 2012 when the study was closed.

### Statistical analyses

Statistical analyses were done at the DMCC. The capacities for disease duration, patient age, and genotype (mutant and normal repeat lengths) to predict SARA scores in patients with SCA were determined by linear regression analysis. Comparisons between SCAs were done by ANOVA and Student t-test for parametric datasets. SARA scores were correlated with the SCAFI-AB, 9HPT, T25FW, UHDRS IV, SF36 and EQVAS data using Pearson correlations and linear regression analyses. Correlations of the SARA score with the Patient Global Impression and the Physician Global Impression were assessed using Spearman’s rank correlation coefficient. Significance of SARA scores by the Patient Global Impression and the Physician Global Impression was assessed using the Kruskal-Wallis test. The frequency of observations in different SCAs was analyzed by chi-square test and Fisher’s exact test, depending on sample size. Mixed modeling, adjusting for repeated measurements, was used to estimate linear trends in SARA, T25FW, functional stage and 9HPT scores overtime. All tests were two-sided and p < 0.05 was considered significant after adjusting for multiple comparisons.

## Results

### Genotypes

Genotyping was confirmed in DNA samples from 287 of the 345 subjects, including 50 SCA1, 60 SCA2, 111 SCA3 and 66 SCA6 patients. First, quality control was ascertained. There was 100% concordance of the first and second multiplex genotype which was performed in 10% randomly chosen samples. Sequence analysis showed 98.8% concordance with repeat number determined by fragment sizing; the discordance by 1-3 repeats was found in seven samples with long SCA3 alleles. Four additional samples (1.4%) showed no or erroneous SCA1, 2, 3 or 6 expansion. The allele size differed from the commercial testing result in 38% of samples by 1 repeat, 10% by 2 repeats and 6% by ≥3 repeats, suggesting the necessity of independent repeat size determination for precise genotyping in these SCAs.

#### Cross section data

A total of 345 patients, including 60 SCA1 (17.4%), 75 SCA2 (21.7%), 138 SCA3 (40.0%) and 72 SCA6 (20.9%) were enrolled in the study from April 2010 to August 2012. The proportion of SCA3 (40.0%) patients in the CRC-SCA cohort is larger and the proportion of SCA2 (21.7%) is smaller than those in the European study population (SCA1: 117, 22.2%; SCA2: 163, 31.0%; SCA3: 139, 26.4%; SCA6: 107, 20.3%; p < 0.001 chi-square) [11]. The distributions of age, sex, age at onset, age at baseline, duration of the disease and SARA Total Score at the baseline of patients with each SCA are listed in Table 1. SCA6 patients had significantly later onset of the disease than those with SCA1, 2 or 3 (p < 0.001). The male:female ratio of patients in each SCA was less than 1.00, but did not significantly differ from 1.00 that is expected from the autosomal dominant inheritance. In 287 patients CAG repeat alleles were determined and the genetic diagnosis was confirmed at the University of Utah. The size of the expanded allele showed a significant inverse correlation with the age at onset in patients with SCA1 (r = −0.608 p < 0.001), SCA2 (r = −0.449 p = 0.008), SCA3 (r = −0.690 p < 0.001), and SCA6 (r = −0.394 p < 0.001). However, the rate of increase of the SARA Total Score, which was estimated from the baseline SARA score and the duration of the disease, showed no correlation with the CAG repeat size in any of the SCAs.

**Table 1:** Baseline clinical characteristics and the increase in SARA Total Score estimated from the baseline score and the duration of the disease

			**Age at onset (yrs)**		**Age at baseline (yrs)**		**Duration of disease (yrs)**		**SARA Total Score**		**Annual estimated increase in SARA Total Score**	**Standard error of parameter estimate**	**R**^**2**^	**R**
**Type of SCA**	**N**	**M/F**	**Mean**	**STD**	**Mean**	**STD**	**Mean**	**STD**	**Mean**	**STD**				
SCA1	60	0.79	40.41	11.40	49.28	12.64	8.93	6.89	14.16	8.39	1.2385	0.0728	0.7176	0.8471
SCA2	75	0.72	36.85	11.69	49.81	13.29	13.46	8.24	16.82	7.46	1.0367	0.0445	0.7882	0.8878
SCA3	138	0.93	39.25	11.81	50.34	12.04	10.92	7.32	14.98	8.78	1.1394	0.0402	0.7490	0.8654
SCA6	72	0.85	52.18	10.34	63.94	10.71	11.76	10.35	14.13	7.64	0.8575	0.0472	0.6980	0.8354
*All*	*345*	*0.84*	*41.66*	*12.66*	*52.92*	*13.40*	*11.30*	*8.28*	*15.07*	*8.23*	1.0471	0.0241	0.7344	0.8570

The mean onset of gait problems was earlier than the onset of speech problems by 4.26 years in SCA1, 3.95 years in SCA2, 5.44 years in SCA3 and 5.08 years in SCA6, and the onset of hand coordination problems by 3.77 years in SCA1, 4.07 years in SCA2, 5.66 years in SCA3 and 3.07 years in SCA6. Overall, the onset of gait problems was significantly earlier than the onset of speech (p < 0.001) or hand coordination (p < 0.001) problems, while the onset of speech showed no significant differences from the onset of hand coordination problems.

**Figure 1:**
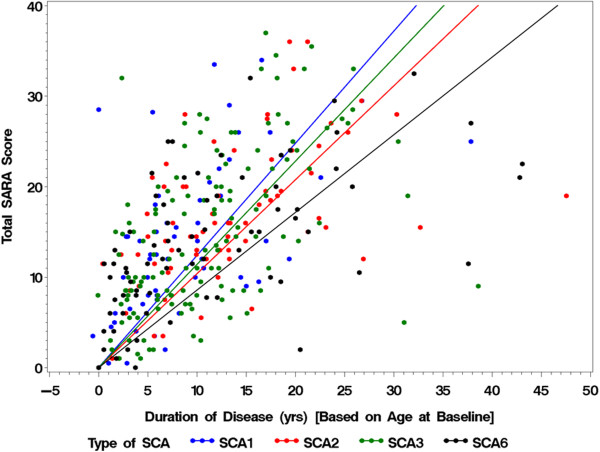
**The SARA Total Score at the baseline and the duration of the disease.** The SARA Total Score (Y axis) plotted against the duration of disease (years; X axis) was calculated from the age at the baseline visit and the age at onset for each subject. Blue dots: SCA1 subjects, red dots: SCA2 subjects, green dots: SCA3 subjects, and black dots: SCA6 subjects. The slope of the color-coded linear regression line indicates the estimated average increase rate of the SARA Total Score of the SCA group.

The annual increase of the SARA Total Score was estimated from the SARA Total Score and the duration of disease at baseline in each SCA, assuming that SARA Total Score was 0 at the disease onset (Table 1). The estimated annual increase of SARA Total Score was the greatest (1.24 ± 0.07, mean ± SE) in SCA1 and significantly (about 20%) greater than SCA2 (1.04 ± 0.04), SCA3 (1.14 ± 0.04) and SCA6 (0.86 ± 0.05) (Figure 1) (p = 0.003). The estimated increase of the SARA Total Score showed no significant correlation with the number of the corresponding expanded CAG repeats in SCA1 (r = 0.049, p = 0.740), SCA2 (r = 0.257, p = 0.053), SCA3 (r = 0.009, p = 0.928) or SCA6 (r = −0.123, p = 0.326).

Patients with SCA2 had greater difficulties with sitting than patients with SCA1 (p = 0.015) and SCA6 (p = 0.002) and with speech than SCA1 (p = 0.042), SCA3 (p = 0.001) and SCA6 (p = 0.024) patients (Table 2). A total of 287 patients completed two trials of 9HPT by both hands; the remaining 58 patients could not complete the entire 9HPT routine due to physical inability, excessive fatigue or unwillingness to complete the test. Patients with SCA2 (n = 60) showed greater difficulties with 9HPT than SCA1 (n = 45, p = 0.002), SCA3 (n = 118; p < 0.001) and SCA6 (n = 65; p = 0.013) patients (Table 2). T25FW was performed twice in 266 patients; of the remaining 79 patients, 73 could be tested neither for the first nor second trial, and six patients were unable to be tested for the second trial due to physical inability, excessive fatigue or unwillingness to walk. The T25FW score (average of two trials) did not significantly differ across SCA1, 2, 3 and 6 (p = 0.271). The above comparisons of difficulties of upper and lower body functions among different SCAs suggest that SCA2 patients have more upper body ataxia than gait, stance and T25FW.

**Table 2:** SARA subscores at baseline

		**SARA**									**T25FW (seconds)**		**9HPT dominant hand (seconds)**		**9HPT non-dominant hand (seconds)**	
		**Total Score**	**Gait**	**Stance**	**Sitting**	**Speech**	**Finger chase**	**Nose finger**	**Alt hand move**	**Heel shin**						
**Type of SCA**	**N**	**Mean**	**Mean**	**Mean**	**Mean**	**Mean**	**Mean**	**Mean**	**Mean**	**Mean**	**N**	**Mean**	**N**	**Mean**	**N**	**Mean**
SCA1	60	14.16	3.49	2.40	0.60	1.89	1.29	1.19	1.52	1.78	50	13.01	49	44.46	44	43.33
SCA2	75	16.82	3.95	2.79	0.99	2.31	1.48	1.51	1.86	1.94	55	13.97	60	54.40	60	59.54
SCA3	138	14.98	4.15	2.80	0.77	1.74	1.22	1.07	1.54	1.69	99	12.36	120	41.06	118	42.98
SCA6	72	14.13	3.94	2.39	0.40	1.88	1.31	1.04	1.39	1.79	62	15.22	69	42.63	65	45.76
*All*	*345*	*15.07*	*3.95*	*2.64*	*0.71*	*1.92*	*1.31*	*1.18*	*1.58*	*1.78*	*266*	*13.48*	*298*	*44.67*	*287*	*47.13*

#### Longitudinal changes in the SARA scores

**Figure 2:**
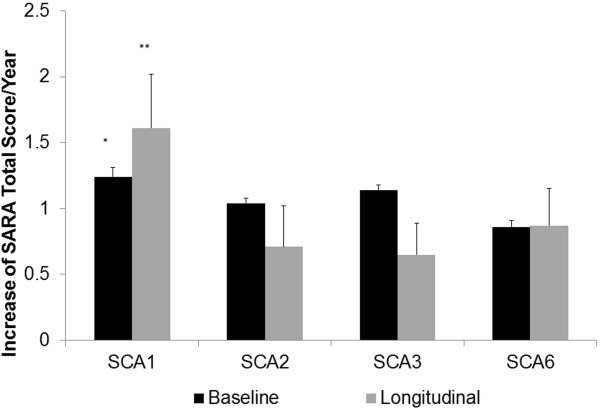
**Progression rate of SCA1, 2, 3 and 6.** The annual rate of increase of the SARA Total Score is shown. Black bar: Annual rate of increase of the SARA Total Score was estimated from cross-section SARA scores and the durations of the disease at the baseline visit. Grey bar: The observed rate of increase of the SARA Total Score during the longitudinal study. (*: p = 0.003, **: p = 0.049, compared with SCA2, SCA3 and SCA6).

The longitudinal data of the SARA Total Score were analyzed to determine the disease progression rate per year of each SCA type using linear regression. Of the 345 patients, 14 (2 SCA1, 4 SCA2, 3 SCA3 and 5 SCA6 subjects) were followed for 24 months, 71 (5 SCA1, 17 SCA2, 30 SCA3 and 19 SCA6 subjects) for 18 months, 73 (16 SCA1, 12 SCA2, 27 SCA3 and 18 SCA6 subjects) for 12 months and 80 (16 SCA1, 19 SCA2, 33 SCA3 and 12 SCA6 subjects) for 6 months. The remaining 107 had only the baseline visit. One or two visits were missed by 14 patients during the follow-up period, including one subject who was followed for 24 months, 7 patients followed for 18 months and 6 patients followed for 12 months. To assess the disease progression rate, the slope of the linear regression of SARA Total Score versus the duration of time on-study (when measurements were obtained) was estimated using mixed modeling adjusting for repeated measurements. On average, the SARA Total Score increased 1.00 ± 0.15 (mean ± SE, p < 0.001) points per year for all SCA patients. The annual rate of change in SARA Total Score was 1.61 ± 0.41 (p < 0.001) points per year in patients with SCA1, 0.71 ± 0.31 (p = 0.024) points per year with SCA2, 0.65 ± 0.24 (p = 0.006) points per year with SCA3, and 0.87 ± 0.28 (p = 0.002) points per year with SCA6 (Figure 2). The observed annual change in SARA Total Score in patients with SCA1 was significantly greater than the annual change in patients with SCA2, 3 and 6 (p = 0.049). Changes in SARA Total Score, Functional Stage, EQ-5D (EQVAS; Health State Indicator) scores, and abbreviated SCAFI (SCAFI-AB) were correlated with the Patient Global Impression and the Physician Global Impression 6 months after the baseline visit in Table 3. These changes were significantly worse in the “Worse” group than in the “Stable/Same” group and the “Improved/Better” group of the Patient Global Impression (p = 0.019 for SARA Total Score, p = 0.011 for Functional Stage, and p = 0.045 for SCAFI-AB) except for EQ-5D (p = 0.561). For changes of the Physician Global Impression only Sara Total Score (p < 0.001) and Functional Stage (p = 0.040) were significantly different between these groups.

**Table 3:** Change in the SARA Total Score, functional stage, EQ-5D and SCAFI-AB from baseline to six-month visit

**Global patient impression**	**SARA Total Score**			**Functional stage**			**EQ-5D**			**SCAFI-AB**		
	**N**	**Mean**	**STD**	**N**	**Mean**	**STD**	**N**	**Mean**	**STD**	**N**	**Mean**	**STD**
Stable	83	0.06	2.61	86	0.02	0.45	85	2.52	20.88	73	−0.02	0.39
Worse	132	0.95	2.64	132	0.21	0.51	129	−3.30	23.02	112	−0.09	0.34
Better	7	−0.86	2.06	7	−0.14	0.24	6	−3.50	18.43	6	0.18	0.48
*All*	*222*	*0.56*	*2.65*	*225*	*0.13*	*0.49*	*220*	*−1.05*	*22.20*	*191*	*−0.06*	*0.36*
**Global physician impression**	**SARA Total Score**			**Functional stage**			**EQ-5D**			**SCAFI-AB**		
	**N**	**Mean**	**STD**	**N**	**Mean**	**STD**	**N**	**Mean**	**STD**	**N**	**Mean**	**STD**
Stable	103	0.18	2.33	108	0.04	0.46	105	−1.46	20.19	95	0.01	0.30
Worse	106	1.18	2.58	105	0.22	0.50	100	−2.13	23.68	88	−0.14	0.42
Better	14	−1.63	2.89	14	0.07	0.47	14	12.29	22.38	9	−0.06	0.33
*All*	*223*	*0.54*	*2.58*	*227*	*0.13*	*0.49*	*219*	*−0.89*	*22.16*	*192*	*−0.06*	*0.37*

Larger numbers reflect worse disease severity in the SARA Total Score and Functional Stage while the reverse applies to EQ-5D and SCAFI-AB.

In the 287 patients in whom the CAG repeat was analyzed, the expansion size showed no correlation with the annual increase of the SARA Total Score estimated from the baseline data in any of the SCAs we studied. However, the observed annual increase of the SARA Total Score showed significant correlation with the age at baseline in SCA2 (r = −0.48, p = 0.006), SCA3 (r = −0.26, p = 0.042) and SCA6 (r = 0.30, p = 0.049) but not in SCA1 (r = −0.16, p = 0.50). The speech SARA score decreased slightly (−0.036 points per year) in patients with SCA2 whereas it worsened in patients with SCA1 (0.409 points per year), SCA3 (0.181 points per year) and SCA6 (0.196 points per year) (p < 0.001).

#### Longitudinal changes in functional stage

The functional stage worsened in patients with all SCAs studied. The rate of worsening significantly differed between patients with different SCA types. Patients with SCA1 showed the fastest worsening of the functional stage with an average increase of 0.411 ± 0.07 points per year, and this was significantly greater than the increase of 0.149 ± 0.05 points in patients with SCA2, 0.181 ± 0.04 points with SCA3 and 0.188 ± 0.05 points with SCA6 (p = 0.002). There were no differences in the worsening rate among SCA2, SCA3 and SCA6.

#### Longitudinal changes in timed measures

The average rate of change in the T25FW score in patients with SCA6 was 6.46 ± 2.09 seconds per year, which was greater than −0.41 ± 2.81 per second per year with SCA1 (p = 0.051), −2.09 ± 2.18 seconds per year with SCA2 (p = 0.005) and 0.02 ± 1.83 second per year with SCA3 (p = 0.021). The average rate of change in the 9HPT score did not differ by SCA types; 3.6 seconds per year in patients with SCA1 per year, 4.4 seconds per year with SCA2, 7.1 seconds per year with SCA3 and 4.5 seconds per year with SCA6 (p = 0.390). Neither did the mean change of the SCAFI-AB differ by SCA types; -0.082 for patients with SCA1, -0.077 per year with SCA2, -0.143 per year with SCA3 and −0.108 per year with SCA6 (p = 0.238). The rate of the SCAFI-AB change did not significantly correlate with the CAG repeat expansion size.

## Discussion

We have prospectively studied phenotypic characteristics in 345 patients with SCA1, 2, 3 and 6 in the US and estimated the rate of progression of ataxia. The patients enrolled in this study represent the largest group of SCA patients studied in the US. Compared with data in the earlier European study [11], our cohort showed a significantly greater proportion of SCA3 and a smaller proportion of SCA2. This difference of the SCA populations between US and Europe may be attributable to immigration patterns in US. There was variability of SCA types between sites, which may be due to settling patterns of immigrants and tertiary referral patterns. The age at onset is variable in all SCAs, but SCA6 mostly began between ages of 50 to 60 years, whereas SCA1, 2 and 3 have an onset predominantly between 30 and 40 years, confirming the previous European data [11].

The annual increase of the SARA Total Score in each SCA was in good agreement between the rate estimated from the cross-section data and the rate observed in the longitudinal study in our US population (Figure 2). SCA1 patients showed a faster increase of the SARA Total Score than patients with SCA2, 3 and 6 in our study. The worsening of the Functional Stage was also significantly faster in SCA1 than three other SCAs, suggesting that SCA1 progresses fastest among the four most common SCAs. The published European data [11] also suggested that patients with SCA1 had faster progression than patients with SCA2, 3 and 6. The rate of increase in our patients with SCA6 appears greater than that in the European population. It should be noted that our patients were evaluated every 6 months in contrast to the annual evaluation in the European study. However, because many patients in our longitudinal study did not completed the follow-up visits at the time the NIH funding ended.

We included patients whose follow-up period was shorter than 2 years in most subjects whereas all patients in the European study completed the 2-year follow-ups. Variability of the progression rate was smaller in the retrospective estimation than in the prospective analysis in our series. This may point to some minimal duration of follow-up that is needed to come to stable calculations of the progression rate in the prospective study.

Although the mean progression rate appears to be faster in the European patients with SCA1, 2 or 3, it did not significantly differ from the progression rate of our cohort. Increasing the number of study subjects and the duration of follow ups in our series could determine whether these are important differences to be considered in designing clinical trials. The male:female ratio of our cohort was 0.84, but this did not significantly differ from 1.00, and there was no significant difference between genders in the rate of increase of the SARA Total Score from either retrospective estimate or prospective longitudinal data in any SCA type. Longitudinal progression studies in patients with SCAs have been conducted in Taiwan [15] and Brazil [16], where progression characteristics were similar to our series.

We defined the age at which an abnormality was noted during usual walking as the age at onset. The age at onset could be even earlier if we defined it as the onset of any ataxic symptom or sign. However, because of greater inaccuracy of the recollection in those events compared with that of gait disturbances, we decided to use the gait as the mode of onset to judge the age at onset. Also we could not be certain that some symptoms, such as blurred vision, were not due to age-related non-ataxic ocular problems. Furthermore, our data clearly showed that gait abnormalities are the mode of onset in most patients.

The number of the CAGs in the expanded (mutant) allele showed a significant inverse correlation with the age at onset in SCA1, 2, 3 and SCA6. However, the CAG expansion size showed no correlation with the rate of increase of the SARA Total Score in patients with SCA1, 2, 3 and 6 in our patients while other studies showed mixed results [11,15-18]. The lack of correlation between the CAG repeat expansion size and the disease progression rate may suggest the presence of proportionally greater confounding factors after the onset of the disease.

Patients with SCA2 showed severe upper body ataxia, such as speech, sitting stability and 9PHT. Patients with SCA2 also showed frequent and prominent postural and kinetic tremor enhanced by intention. Although the severely impaired upper limb coordination in our SCA2 patients may at least be partly attributable to tremor, tremor could not fully account for severe impairments of speech and sitting balance. Thus, our data suggest that SCA2 affects motor coordination in the upper body more severely than that in the lower body. However, neither the observed rate of increase in the upper body SARA score nor that in the lower body SARA score was significantly different between SCA2 and other SCAs. SCA2 may have disproportionally fast progression of upper body ataxia in an early phase of the disease compared with SCA1, 3 and 6.

As in the European study, the EQVAS score showed no correlation to SARA scores in our patients. SCA-specific measures of the quality of life are needed. However, SF36 is widely used for various disorders, and non-pain scores of SF36 may give a better correlation with SARA scores.

None of the timed measures (T25FW, 9HPT and SCAFI-AB) captured the faster progression of ataxia in SCA1 than SCA2, 3 and 6. In T25FW, the devices used to assist walking complicate the measurements. As in the PATA test, results of these timed measures tend to vary and this raises some doubt regarding the value of composite measures, such as SCAFI and SCAFI-AB.

## Conclusions

We conclude that disease progression rates in patients with SCA1, 2, 3 and 6 are likely to be comparable between US and Europe. International collaborative clinical studies, including therapeutic trials, may be feasible.

## Abbreviations

CAG: Cytosine-adenine-guanine; CEPH: Centre d’Etude du Polymorphisme Humain; CRC-SCA: Clinical research consortium for spinocerebellar ataxias; DMCC: RDCRN Data monitoring coordinating center; EQVAS: EQ-5D (quality of life) visual analogue scale; EQ-5D: European quality of life-5 dimensions; polyQ: Polyglutamine; RDC: Rare disease consortia; RDCRN: Rare disease clinical research network; SARA: Scale for the assessment and rating of ataxia; SCA: Spinocerebellar ataxia; SCAFI: SCA Functional index; SCAFI-AB: Abbreviated SCA functional index; T25FW: Timed 25-ft walk test; UHDRS IV: Unified Huntington’s disease rating scale functional assessment; US: The United States; 9HPT: 9-hole pegboard test.

## Competing interests

Dr. Ashizawa was supported by NIH (RC1NS068897) for this work (2009-2012) and is receiving another NIH grant (R01NS083564) (2013-2018). He also receives research funding from the National Ataxia Foundation (2013) and the Muscular Dystrophy Association (2013-2015). He has been receiving royalty from Baylor College of Medicine (since 2001). He has received travel reimbursement from the Japanese Society of Neurology (2013), Cooperative Clinical Research Network–Friedreich Ataxia (2013), Muscular Dystrophy Foundation (2013), Central China University (with honorarium, 2012), Baylor College of Medicine (2012), Texas Neurological Society (2012), Unstable Microsatellites and Human Diseases (2011), Fudan University (with honorarium, 2011) and International Myotonic Dystrophy Consortium (2011).

Ms. Figueroa reports no disclosures.

Dr. Perlman received research grantS from Santhera Pharmaceuticals (2011), EDISON PHARMACEUTICALS (2012-13), FRIEDREICHS ATAXIA RESEARCH ALLIANCE (2002-2013), and National Ataxia Foundation (2013).

Dr. Gomez receives NIH grant R01NS033202 (2010-2015).

Dr. Wilmot is a member of the Data Safety Monitoring Board for Santhera Pharmaceuticals and has received support from the Cooperative Clinical Research Network–Friedreich Ataxia.

Dr. Schmahmann reports no disclosures.

Dr. Ying has been supported by NIH grants R21 NS059830, R01 EY019347, R01 NS056307, R21 EY022150, and received other research support from the Brain Science Institute and 5RC1NS068897.

Dr. Zesiewicz Dr. Zesiewicz received compensation from UCB Pharma, Teva and GE for Speaking activities, grants from Astellas Pharmaceuticals, Baxter, Friedreich’s Ataxia Research Alliance, Takeda, Edison Pharmaceuticals, and GlaxoSmithKline for the past yer.

Dr. Paulson is funded by NIH grants R01NS038712 (2009-2013), R01AG034228 (2009-2013), and R03NS072967 (2011-2013), has received license fee payments from Sirna Therapeutics (2007-2011), and received research contract support from Shire Human Genetic Therapy (2010-2012).

Dr. Shakkottai receives NIH grant K08NS0.72158 (2010-2015), funding through the Dystonia Medical Research Foundation.

Dr. Bushara reports no disclosures.

Dr. Kuo receives NIH grant K08NS083738 (2013-2018) and Parkinson’s Disease Foundation CEI-1241. He also received Louis V Gerstner Jr. Scholar Award, and American Academy of Neurology research fellowship.

Dr. Geschwind receives grant funding from NIH/NIA ROI AG-031189 for his work on the early diagnosis of human prion disease, NIH/NIA AG031220, P01 Ago21601, and the Michael J. Homer Family Fund. Dr. Geschwind is a consultant for Lundbeck, Inc, MedaCorp, The Council of Advisors and Neurophage. Dr. Geschwind’s CJD-RPD research, not salary, is also supported by the UCSF MAC ADRC P50AG023501, NIH/NCRR UCSF-CTSI Grant Number UL1 RR024131, NIH/NIA AG021601, and NIH/NINDS Contract N01-NS-0-2328. Dr. Geschwind’s Huntington Disease research is supported by CHDI, Inc and NIH/NINDS (NS40068).

Dr. Xia receives funding from Acorda Pharmaceutical (2013).

Dr. Mazzoni reports no disclosures.

Dr. Krischer receives NIH grant U54 NS064808 (2009-2014)

Mr. Cuthbertson reports no disclosures.

Ms. Roberts Holbert reports no disclosures.

Dr. Ferguson reports no disclosures.

Dr. Pulst receives grants from NIH (RC4NS073009; R21NS079852; R21NS081182, and RO1RO1NS33123; 2010-2015) and the National Ataxia Foundation (2013).

Dr. Subramony receives honoraria and travel reimbursements from Athena diagnostics; compensation from Elsevier Co. for editing Handbook of Clinical Neurology, and received grants from National Ataxia Foundation (2011, 2012, 2013).

## Authors’ contributions

All authors have made substantial contributions to conception and design, or acquisition of data, or analysis and interpretation of data; have been involved in drafting the manuscript or revising it critically for important intellectual content; and have given final approval of the version being submitted. TA participated in designing and conceptualizing the study, collecting data, analyzing and interpreting the data, drafting the manuscript and revising the manuscript. He was the principal investigator for the entire research operation of the CRC-SCA. KF participated in collecting data, analyzing and interpreting the data, drafting the manuscript and revising the manuscript. SP participated in collecting data, analyzing and interpreting the data, and revising the manuscript. CG participated in collecting data, analyzing and interpreting the data, and revising the manuscript. GW participated in collecting data, analyzing and interpreting the data, and revising the manuscript. JS participated in collecting data, analyzing and interpreting the data, and revising the manuscript. SHY participated in collecting data, analyzing and interpreting the data, and revising the manuscript. TZ participated in collecting data, analyzing and interpreting the data, and revising the manuscript. HP participated in collecting data, analyzing and interpreting the data, and revising the manuscript. VS participated in collecting data, analyzing and interpreting the data, and revising the manuscript. KB participated in collecting data, analyzing and interpreting the data, and revising the manuscript. S-HK participated in collecting data, analyzing and interpreting the data, and revising the manuscript. MG participated in collecting data, analyzing and interpreting the data, and revising the manuscript. Dr. Xia participated in collecting data, analyzing and interpreting the data, and revising the manuscript. PM participated in collecting data, analyzing and interpreting the data, and revising the manuscript. JK participated in designing and conceptualizing the study, analyzing and interpreting the data, and revising the manuscript. He processed all data generated and placed them in the DMCC database. DC, a statistician at the DMCC, conducted all statistical analyses under the supervision of Jeffrey Krischer, Ph.D., Director of the Data Management Coordinating Center (DMCC) of the NIH Rare Disease Clinical Research Network. ARH participated in analyzing and interpreting the data, and revising the manuscript. With Dr. Ashizawa, Dr. Subramony and Dr. Pulst, she designed all case report forms and managed the DMCC database. JF participated in analyzing and interpreting the data, and revising the manuscript. SP participated in designing and conceptualizing the study, collecting data, analyzing and interpreting the data, drafting the manuscript and revising the manuscript. He was responsible for the DNA analysis and data interpretation. SHS participated in designing and conceptualizing the study, collecting data, analyzing and interpreting the data, drafting the manuscript and revising the manuscript. He was the project leader of the natural history study of the CRC-SCA.
